# Association between body weight perception and actual body mass index among adult women in Erbil city, Iraq

**DOI:** 10.1186/s41043-024-00512-8

**Published:** 2024-01-29

**Authors:** Sherzad A. Shabu, Mariwan H. Saka, Manhal N. Boya, Hamdia M. Ahmed, Sahar M. Zaki, Florentina Hettinga, Nazar P. Shabila

**Affiliations:** 1 Department of Community Medicine, College of Medicine, Hawler Medical University, Erbil, Kurdistan Region Iraq; 2 Department of Medicine, College of Medicine, Hawler Medical University, Erbil, Kurdistan Region Iraq; 3 College of Physical Education, University of Salahaddin, Erbil, Kurdistan Region Iraq; 4 College of Health Sciences, Hawler Medical University, Erbil, Kurdistan Region Iraq; 5 Department of Sport, Exercise, and Rehabilitation, Northumbria University Newcastle, Newcastle upon Tyne, UK; 6 College of Health Sciences, Catholic University in Erbil, Erbil, Kurdistan Region Iraq

**Keywords:** Body weight, BMI, Perception, Consistency

## Abstract

**Background:**

The misperception of body weight can significantly affect individuals' health behaviors, such as physical activity, diet, and weight management. This study aimed to examine the association between body weight perception and actual body mass index (BMI) among adult women and explore the factors influencing this relationship.

**Methods:**

Five hundred forty female individuals aged 18–65 participated in this cross-sectional study. The validated Global Physical Activity Questionnaire was used for data collection. The BMI of the participants was calculated from measured body weight and height. Body weight perception was assessed using a single questionnaire item. The association of BMI and body weight perception was assessed, and the result was categorized as underestimation, consistency, and overestimation. The Chi-square test was used to assess the association between the consistency of BMI and body weight perception by different sociodemographic factors. The kappa test was used to analyze the consistency of BMI and body weight perception.

**Results:**

Of the 540 participants, 13.3% underestimated their body weight status, 79.1% accurately perceived their body weight status, and 7.6% overestimated their body weight status. Unmarried women (11.7%) were more likely than ever married (4.3%) to overestimate their body weight (*p* = 0.005). On multiple logistic regression, being unmarried (OR = 1.68 (95%CI 1.01–2.80)) was significantly associated with body weight misperception. Body weight perception and BMI categories showed a significantly good consistency (kappa = 0.612, *p* < 0.001). Correct perception of body weight was highest among the overweight, followed by normal weight and underweight individuals (82.1%, 75.8%, and 72.2%, respectively).

**Conclusion:**

Body weight perception was well associated with actual body weight status. Unmarried women are more likely to misperceive body weight, particularly overestimating it. Underestimation of body weight was relatively high and much higher than the overestimation, which might keep obese individuals from weight loss activities. Preventing obesity should include awareness about body weight misperceptions.

## Background

Obesity is considered an important public health problem and a significant risk factor for many physical and mental health problems, such as diabetes mellitus, cardiovascular diseases, and depression [1]. Body mass index (BMI), commonly used to identify or classify obesity, is calculated as the body mass in kilograms divided by height in meters squared [2]. Body weight perception is the subjective evaluation of one's own body weight and shape, which may not always align with the actual BMI [3]. Research has shown that misperceptions of body weight can significantly affect individuals' health behaviors, such as physical activity, diet, and weight management [4]. This misperception is particularly important when underestimating body weight since obese individuals might refrain from diet restriction and weight loss activities, thinking they are of normal weight. It might contribute to the increasing prevalence of overweight and obesity and their associated health consequences at individual and population levels [4, 5].

It has been shown that women, in particular, are more likely than men to perceive themselves as overweight, even when their BMI is within the healthy range [6]. This discrepancy between body weight perception and BMI may lead to unnecessary dieting and weight loss attempts, negatively affecting physical and mental health [5]. Underestimation of body weight in obese or overweight individuals might result in denial or minimization of current body weight being a health risk. It can lead to an increase in obesity-associated health problems [7]. Therefore, body weight perception is recognized as a strong determinant of nutritional habits and weight management practices [8]. This is in addition to the fact that body dissatisfaction is always related to some adverse psychological consequences such as anxiety and depression [9].

Several studies have investigated the association between body weight perception and actual BMI among women, but the results have been inconsistent [10]. The results of some studies have revealed that women who perceive themselves as overweight are more likely to have a higher BMI. In contrast, others have found no association between body weight perception and BMI or misconception of body weight [5, 11, 12]. Some studies have reported that women who perceive themselves as overweight are more commonly involved in weight loss behaviors, while others have found no association [13, 14]. On the other hand, other studies have found that obese women who perceive themselves as normal are at risk of doing nothing while they need to reduce body weight [7].

Understanding the relationship between body weight perception and actual BMI among women is important for developing effective interventions to prevent and manage overweight and obesity [15]. This study aimed to examine the association between body weight perception and actual BMI among adult women and explore the factors influencing this relationship. The results of this study will help the current understanding of body weight perception and its impact on health behaviors among women and direct the development of targeted measures to improve body weight perception and promote healthy weight management.

## Methods

### Design and setting

This cross-sectional study was conducted in Erbil, Iraqi Kurdistan Region, from November 2022 to February 2023.

### Participants

The sample size was calculated using the Epi-info, assuming that the prevalence of correctly perceiving body weight among adult women is 70.1% [16] with a 95% confidence interval for the prevalence and ± 5% precision. A sample size of 522 adult women was calculated and increased to 600 to account for non-response.

A convenience sample of female individuals aged 18–65 from Erbil city was invited to participate in the study. The sample was selected with the help of the Center for Research and Education in Women's Health of Hawler Medical University. The center works with different female groups in the community by providing health awareness and education services. The study sample was selected from the network of this center, which is in contact with other women's groups. The sample was selected from these groups of women.

### Instruments and data collection

The researchers designed a questionnaire to collect sociodemographic information, such as age, marital status, region of residence, education level, job, and self-assessed economic status. Information was also collected about the use of social media and body weight perception.

The body weight perception was measured using a question with the three response options of underweight, normal weight, and overweight based on the participants’ visual/imaginary perception. After obtaining information about body weight perception, using standardized protocols, each participant's height and body weight were measured by the researchers at the Center for Research and Education in Women's Health or at the workplace.

Professional physician-grade digital scales were used to collect weight data. The scale was placed on firm flooring. The participants were asked to remove shoes and heavy clothing such as sweaters and stand with both feet in the center of the scale. The weight was recorded to the nearest decimal fraction (e.g., 60.1 kg). The height was measured using a portable stadiometer. The participant was asked to remove the shoes and stand on the wall with feet flat, legs straight, arms at sides, and shoulders level, and looking straight ahead. The height was recorded to the nearest 0.1 cm.

The BMI was calculated by body weight divided by height squared (kg/m^2^). The BMI was classified into three groups: underweight (BMI < 18.5 kg/m^2^), normal weight (BMI = 18.5–24.9 kg/m^2^), and overweight (BMI ≥ 25 kg/m^2^) [2].

The validated Global Physical Activity Questionnaire (GPAQ) was used to assess the participants' physical activity performance [17, 18]. The cutoffs in the Global Physical Activity Questionnaire analysis guide were used for physical activity assessment [18]. Thus, performing regular physical activity included engagement in at least 20 min of vigorous intensity activity per day on at least three days per week, 30 min of moderate-intensity activity or walking per day on at least five days per week, or a combination of both.

### Ethical aspects

This study was reviewed and approved by the Research Ethics Committee of Hawler Medical University. Written informed consent was obtained from the participants before participation in the study. The study was conducted according to the principles of the Helsinki Declaration.

### Data analysis

The statistical package for the social sciences (SPSS, version 22.0) was used for data analyses. The outcome of comparing body weight perception and BMI was divided into three categories: (1) underestimation, (2) consistency, and (3) overestimation. Mean + SD for numerical data and frequencies for categorical data were used to present the findings. The Chi-square test was used to test the association between proportions. Analysis of variance analysis (ANOVA) was used to compare means. The Chi-square test was used to assess the association between the consistency of BMI and body weight perception by different sociodemographic factors. The kappa test was used to analyze the consistency of BMI and body weight perception. A *P* value of ≤ 0.05 was considered statistically significant for all the associations. Multiple logistic regression was also used to control for the sociodemographic factors of the participants. Adjusted odds ratios (OR) and 95% confidence intervals were calculated. The binary outcome for logistic regression included misperception of body weight (combining underestimation and overestimation).

## Results

The mean age of the 540 participants was 34.3 ± 12.6 years. The mean BMI was 26.1 ± 6.4. The demographic and lifestyle characteristics of the participants are given in Table 1. For the actual BMI groups, 3.3% were underweight, 42.8% were normal weight, and 53.9% were overweight. For body weight perception, 6.1% perceived themselves as underweight, 43% as normal weight, and 50.9% as overweight. Regarding the association of BMI and body weight perception, 13.3% of the participants underestimated their body weight status, 79.1% correctly perceived their body weight status, and 7.6% overestimated their body weight status. Marital status was significantly associated with the consistency of BMI and perceived body weight (*p* = 0.005). Overestimation of body weight was more common among unmarried (11.7%) than ever married (4.3%). There was no significant difference between other variables in the consistency of BMI and body weight perception. The details of body weight perception and BMI consistency according to the demographic and lifestyle characteristics of the participants are given in Table 1 and 2.

**Table 1:** Details of body weight perception and BMI consistency according to sociodemographic characteristics

Characteristic	Body weight perception						Total		*χ2*	P*
	Underestimation		Consistency		Overestimation					
	N	(%)	N	(%)	N	(%)				
*Age group (years)*										
18–30	26	(10.6)	194	(79.2)	25	(10.2)	245	(45.4)	7.4	0.115
31–40	19	(15.0)	99	(78.0)	9	(7.1)	127	(23.5)		
> 40	27	(16.1)	134	(79.8)	7	(4.2)	168	(31.1)		
*Education level*										
High school or less	23	(13.7)	131	(78.0)	14	(8.3)	168	(31.1)	4.2	0.383
College	36	(15.7)	175	(76.4)	18	(7.9)	229	(42.4)		
Postgraduate	13	(9.1)	121	(84.6)	9	(6.3)	143	(26.5)		
Region										
City	63	(12.8)	394	(80.1)	35	(7.1)	492	(91.1)	3.5	0.17
Outside city	9	(18.8)	33	(68.8)	6	(12.5)	48	(8.9)		
*Marital status*										
Ever married	41	(13.6)	247	(82.1)	13	(4.3)	301	(55.7)	10.4	0.005
Unmarried	31	(13.0)	180	(75.3)	28	(11.7)	239	(44.3)		
Job										
Non-manual employee	30	(16.5)	141	(77.5)	11	(6.0)	182	(33.7)	5.9	0.438
High rank professional or managerial	19	(11.7)	131	(80.4)	13	(8.0)	163	(30.2)		
Student	15	(11.8)	98	(77.2)	14	(11.0)	127	(23.5)		
Housewife	8	(11.8)	57	(83.8)	3	(4.4)	68	(12.6)		
*Economic situation*										
Below average and average	50	(13.5)	288	(77.6)	33	(8.9)	371	(68.7)	2.98	0.225
Above average	22	(13.0)	139	(82.2)	8	(4.7)	169	(31.3)		
*Use of social media*										
Sometimes	42	(13.9)	245	(80.9)	16	(5.3)	303	(56.1)	5.3	0.071
Frequently	30	(12.7)	182	(76.8)	25	(10.5)	237	(43.9)		
*Regular physical exercise*										
Doing	11	(13.8)	66	(82.5)	3	(3.8)	80	(14.8)	1.98	0.372
Not doing	61	(13.3)	361	(78.5)	38	(8.3)	460	(85.2)		
All participants	72	(13.3)	427	(79.1)	41	(7.6)	540	(100.0)		

*Chi-square test

On multiple logistic regression, only being unmarried (OR = 1.68 (95%CI 1.01–2.80)) was significantly associated with body weight misperception, as shown in Table 2

**Table 2:** Multiple logistic regression of body weight misperception with sociodemographic characteristics

Characteristic	OR	95% CI for OR		*P*
		Lower	Upper	
*Age group (years)*				
18–30	Ref			
31–40	1.59	0.83	3.06	0.162
> 40	1.65	0.86	3.15	0.130
*Education level*				
High school or less	Ref			
College	1.08	0.52	2.24	0.838
Postgraduate	0.73	0.30	1.75	0.479
*Region*				
City	Ref			
Outside city	1.60	0.82	3.14	0.171
*Marital status*				
Ever married	Ref			
Unmarried	1.68	1.01	2.80	0.044
*Job*				
Non-manual employee	Ref			
High rank professional or managerial	0.99	0.57	1.73	0.983
Student	0.66	0.32	1.35	0.258
Housewife	0.83	0.34	2.04	0.690
*Economic situation*				
Below average and average	Ref			
Above average	0.84	0.51	1.37	0.484
*Use of social media*				
Sometimes	Ref			
Frequently	1.26	0.81	1.97	0.305
*Regular physical exercise*				
Doing	Ref			
Not doing	1.26	0.67	2.38	0.476

Multiple logistic regression

Table 3 provides a consistency analysis of body weight perception and BMI categories. Kappa tests revealed statistically significant consistency in body weight perception and BMI categories (kappa = 0.612, *p* < 0.001). Kappa > 0.6 indicates a good consistency of BMI categories and body weight perception. Correct perception of actual body weight was highest among the overweight, followed by normal weight and underweight individuals (82.1%, 75.8%, and 72.2%, respectively).

**Table 3:** Details of the consistency analysis of body weight perception and BMI categories

Calculated BMI category	Body weight perception						P*
	Underweight		Normal weight		Overweight		
	N	%	N	%	N	%	
Underweight	13	(72.2)	5	(27.8)	0	(0.0)	< 0.001
Normal weight	20	(8.7)	175	(75.8)	36	(15.6)	
Overweight	0	(0.0)	52	(17.9)	239	(82.1)	

*Kappa

The mean ± SD BMI of those with both normal calculated BMI and normal self-perception was 22.4 ± 1.67. The mean ± SD BMI of those in the overweight BMI category but perceive themselves as normal weight was 29.4 ± 14.43. The mean ± SD BMI of those with normal calculated BMI but who perceive themselves as overweight was 23.3 ± 1.06. The mean ± SD BMI of those with normal calculated BMI but who perceive themselves as underweight was 20.8 ± 1.76. Details of the mean BMI of the participants according to their actual calculated BMI and self-perception groups are shown in Table 4.

**Table 4:** Mean body weight of the participants according to their calculated BMI and self-perception

Calculated BMI category	Body weight perception						P*
	Underweight		Normal weight		Overweight		
	Mean ± SD	Median	Mean ± SD	Median	Mean ± SD	Median	
Underweight	17.3 ± 0.95	17.5	17.6 ± 0.55	17.6	NA	NA	< 0.001
Normal weight	20.8 ± 1.76	20.5	22.4 ± 1.67	22.6	23.3 ± 1.06	23.3	
Overweight	NA	NA	29.4 ± 14.43	27.0	29.7 ± 3.54	28.9	

*ANOVA test

NA: not applicable for not having any cases in these categories

## Discussion

Body weight perception strongly determines nutritional habits and weight management practices. Misperception of body weight might result in unnecessary diet restriction and weight loss or increased obesity and associated physical and mental health consequences [7–9]. The actual BMI categories in the current study were 3.3% underweight, 42.8% normal, and 53.9% overweight. In a study conducted in China, the BMI categories were 4.9%, 71.2%, and 23.9%, respectively [19]. In another study conducted in Saudi Arabia in 2021, the proportions were 6.4%, 38.9%, and 53.8%, respectively [4]. The current study also revealed that only 14.8% of the participants exercised regularly, while the Saudi Arabia study showed that 10.2% conducted vigorous physical activity [4].

The current study revealed that 13.3% of the participants underestimated their body weight status, 79.1% accurately perceived their body weight status, and 7.6% overestimated their body weight status. This high rate of consistency might be attributed to the relatively high education level of the studied sample, or the selected sample might be more active in performing physical exercise or more concerned about health. These rates differed from the study conducted in China, in which 27.6% underestimated their body weight status, 67.6% were consistent, and only 4.8% overestimated their body weight status [19]. A study conducted in the Republic of Korea revealed that 3.4% of the participants underestimated their body weight status, 82.6% were consistent, and 14% overestimated their body status [5]. Furthermore, a study conducted in Saudi Arabia has revealed that 42% of the participants have misclassified their body weight status compared to 58% who have properly classified their body weight status [4]. The above differences could be related to different designs, settings, and sample characteristics of the different studies. A study conducted in Texas also concluded that the misperception of body weight and unhealthy body weight-related behaviors are common among adult women [13].

The current study found that body weight perception varied significantly by marital status. Unmarried women were more likely to misperceive their body weight than married women. These results agree with a study conducted in the Republic of Korea, which concluded that body weight misperception was significantly higher among unmarried women than married women [5]. In the current study, consistency of body weight perception was more common in married women, while overestimation was more common in unmarried women. Married and unmarried women in this setting might have different standards of ideal body weight. Research has consistently shown that unmarried women overestimate their body weight more frequently, are dissatisfied with it, and try to lose it [20]. On the other hand, married women in Arabic countries frequently associate overweight with richness, health, strength, and fertility [21].

In the current study, the age group factor was positively but not significantly associated with misperception of body weight. In contrast, a study from Saudi Arabia involving 6,239 male and female participants revealed that older age groups had a significantly more frequent misclassification of their body weight [4]. Moreover, a study conducted on 22,121 adult women in the Republic of Korea in 2022 revealed that the mismatch of body weight was higher among the late adulthood group [5]. The current study might have failed to show any statistically significant association between body weight misperception and the age of the participants due to the relatively small sample size.

This current study revealed an insignificant statistical association between accurate body weight perception and the education level of the participants. This finding contradicts the Saudi Arabia study, which revealed a higher accurate perception with increasing education level [4]. A study conducted in Texas revealed that women with at least some college degrees were less likely to misperceive their body weight than those with less education [13].

According to the current study, an insignificant statistical association was reported between misperception of body weight and the region of residence, with higher misperceptions among those from outside cities. These results were consistent with a study conducted in China, where an insignificant association between body weight misperception and residence (urban or rural) was also reported [19].

The current study revealed an insignificant negative statistical association between body weight misperception and regular physical exercise. A study conducted in the Republic of Korea has also reported a significant decrease in body weight misperception with increased days of walking [5]. These results were also consistent with a study conducted in Saudi Arabia, which revealed that more sedentary participants were more likely to have body weight misperception [4].

In this study, women's use of social media was also insignificantly and positively associated with body weight misperception. Unlike these results, a study conducted in Texas has reported that internet use was significantly associated with less overweight misperception among young reproductive age women [13]. Evidence shows that social media influences one's need to alter self-presentation to fit in with highly pressured societal ideals [22]. Unrealistic and idealized images of body size in social media might noticeably affect body weight perception. Pressure about appearance from social media delivers unrealistic societal standards of physical beauty. Women might use upward social comparisons and feel they do not match the thin ideal [22–24].

The economic situation of the studied participants was insignificantly and negatively associated with body weight misperception. Another study from Malaysia also showed no association between household income and women's perception of body weight [25]. In contrast, a study from the Republic of Korea showed that proper weight perception was positively related to poverty level [26]. Another study from the Republic of Korea also showed that favorable socioeconomic status predicted participants' misperceptions of body weight, especially in women [12]. This comparison shows that the economic situation of women is associated differently with body weight misperception in different populations and settings. The economic situation might not be an independent factor for this association, and other related factors might work as confounders [26].

The high rate of consistency between body weight perception and actual BMI in this study might indicate that women in Erbil have high awareness about their body weight. This high awareness is particularly true for highly educated women, as our sample consisted mainly of them. A higher rate of underestimation than overestimation might indicate that an important segment of women might not be aware of the presence of overweight or obesity and thus obscure the associated health risks.

In the current study, the mean BMI of the participants according to their calculated BMI and self-perception groups showed that the adult women of different groups with the misperception of body weight had a mean BMI close to the BMI range of the calculated BMI category. This indicates that adult women with a misperception of their body weight might not have enough information about what constitutes normal body weight and might not know what BMI is, how it is calculated, and what the normal range for normal weight is. Unfortunately, the availability of such knowledge among the participants was not assessed, and this possible knowledge gap needs to be addressed in future studies. There is limited knowledge of BMI cutoff values among the general population. For example, a study showed that over 80% of people incorrectly define specific BMI levels and their meaning. It also showed that self-awareness of obesity is limited, with only 16% of people aware of their own personal BMI [27].

### Strengths and weaknesses

This study is the first to address body weight misperception in Erbil and Iraq, as no data exist on this important topic in Iraq, particularly on adult women. The calculation of the BMI of the participants was made objectively in this study. This study had several limitations. Firstly, the study included a convenience sample of adult women from only a few settings in Erbil city. The sample cannot truly reflect the entire adult female population, especially as the rural population, those with no or low education, and those with low economic conditions were underrepresented. Therefore, these findings may not be generalizable to all adult female populations in Erbil city or Iraq. Secondly, most sociodemographic and lifestyle characteristics were obtained from self-reported questionnaires and might be inherently biased by the individual's subjective feelings.

## Conclusions

Body weight perception was well associated with actual body weight status among adult women in Erbil city. Body weight perception varied significantly by marital status, with unmarried women more likely to misperceive their body weight, particularly overestimating it. The underestimation of body weight was relatively high and much higher than the overestimation. Such underestimation might result in denial or minimization of current body weight as a health risk among obese individuals. It might keep them from weight loss activities, leading to increased obesity-associated health problems. Therefore, interventions for preventing obesity should also focus on awareness about body weight misperceptions. Further studies are needed to assess body weight perception among less educated women since this group of women was not sufficiently represented in the sample of this study.

## Data Availability

The dataset used for the current study is available in the Mendeley dataset repository (https://doi.org/10.17632/h8gn74m8mr.1).

## Abbreviation

BMI: Body mass index
